# Phase Transition in the Boltzmann–Vlasov Equation

**DOI:** 10.1007/s10955-019-02222-6

**Published:** 2019-01-22

**Authors:** A. C. Fowler

**Affiliations:** 1 MACSI, University of Limerick, Limerick, Ireland; 2 OCIAM, University of Oxford, Oxford, UK

**Keywords:** Phase transition, Boltzmann equation, Stability theory

## Abstract

In this paper we revisit the problem of explaining phase transition by a study of a form of the Boltzmann equation, where inter-molecular attraction is included by means of a Vlasov term in the evolution equation for the one particle distribution function. We are able to show that for typical gas densities, a uniform state is unstable if the inter-molecular attraction is large enough. Our analysis relies strongly on the assumption, essential to the derivation of the Boltzmann equation, that $\nu \ll 1,$ where $\nu =d/l$ is the ratio of the molecular diameter to the mean inter-particle distance; in this case, for fluctuations on the scale of the molecular spacing, the collision term is small, and an explicit approximate solution is possible. We give reasons why we think the resulting approximation is valid, and in conclusion offer some possibilities for extension of the results to finite amplitude.

## Introduction

The application of the Boltzmann equation to the fluid mechanical properties of matter has been extraordinarily successful. The Chapman–Enskog expansion [8] of its solution provides a mechanistic explanation of viscosity and thermal conductivity, and equally Boltzmann’s *H*-theorem provides an explicit basis for the concept of entropy and the laws of thermodynamics.

In this context, one of the intriguing properties of materials is that of phase transition, which on the face of it is inconsistent with the *H*-theorem, which provides a Lyapunov function for the velocity distribution which drives it to a unique (Maxwellian) equilibrium. On the other hand, phase changes correspond to the existence of multiple steady states, as instanced for example by the van der Waals equation, which seems prima facie contradictory to the *H*-theorem. Actually, it is not as straightforward as this, since the equilibrium Maxwellian distribution depends on number density and temperature, and assumes these quantities are spatially uniform; if one allows spatially varying number density, other possibilities can occur, and indeed this is the subject of the present paper.

Of course, the resolution of this apparent conundrum lies in the fact that the *H*-theorem assumes a one particle distribution function $f(\mathbf{r},\mathbf{v},t)$ which is independent of the spatial variable $\mathbf{r}.$ If this restriction is lifted, the possibility of spatial variation arising through an instability occurs, with the interpretation of such an instability being the onset of condensation. It is therefore of interest to examine the stability of the spatially uniform equilibrium of the Boltzmann equation, with a view to understanding how phase transition occurs.

There is, however, nothing in the Boltzmann equation which seems to provide a mechanism for instability; to provide such a mechanism, we will interpret the intermolecular potential as consisting of two parts: a steep repulsive part, which we model as the usual Boltzmann collisional interaction, and a longer range attractive part, and it is this which provides the instability mechanism: attractive forces between molecules induce a tendency to form clusters, and this tendency is enhanced in conditions of high density or low temperature.

A number of authors have investigated this problem. The original approach uses methods of classical statistical mechanics (e. g., Born and Fuchs [4], Mermin [16] and van Kampen [20]), which allow the derivation of the van der Waals equation of state from a virial expansion of the grand partition function (e. g., Schwabl [19], p. 236 ff). Later authors address the problem directly. For example, Grmela [13] enunciates essentially the same problem which is of concern here. And indeed, his approach is similar: he writes down a Boltzmann–Vlasov equation similar to that which we present below, where the reference to Vlasov reflects the additional attractive term which is used in theories of plasma dynamics [22]. But in common with much of the literature on kinetic theory, the presentation is discursive, and the difficulty of dealing with the linearised collision operator prevents any clear conclusion.

Liboff [15] also presents similar stability results to those we derive below, for a range of different attractive power law potentials, but largely ignores the effects of collisions, only modifying his analysis by including a simplified (algebraic) collision term due to Bhatnagar et al. [3], yielding the so-called BGK approximation, which is analytically tractable. A similar approach has recently been adopted by Benilov and Benilov [2].

De Sobrino [10] takes the application of the Boltzmann equation further. Insofar as phase transition can be understood by derivation of the van der Waals equation of state, he shows that by including the two essential constituents of this equation, intermolecular attraction and molecular crowding, in the prescription of the closure for the two-particle distribution function, the van der Waals equation emerges as the equation of state of the equilibrium solutions. Other approaches and related discussions can be found in the papers of Penrose [18], Wisnivesky [21] and Chen [9].

In this paper we consider the problem of condensation from the point of view of kinetic theory, by analysing the Boltzmann–Vlasov equation for the one particle distribution function, which includes the Boltzmann collision integral and the Vlasov term describing the intermolecular attraction. Although the ideas of scale have been adumbrated before, here we explicitly non-dimensionalise the model, and show that the collision term is small, providing the gas is sufficiently rarified, in the sense that $\nu =d/l\ll 1$: here *d* is molecular diameter and *l* is the mean inter-particle spacing; in practice this assumption is justified, except near the critical point. This allows us to derive an explicit condition for instability, much in the manner of Liboff [15]; however, we go further by considering explicitly the corrective rôle of the collision integral, and we show that it is small, although we surmise that this is not the case in the condensed phase equilibrium.

The rest of the paper proceeds as follows. In Sect. 2, we present the Boltzmann equation and its modification by the Vlasov term, and we non-dimensionalise the system, which introduces two important dimensionless parameters: $\nu ,$ as described above, and $\beta ,$ which measures the strength of the inter-molecular attraction. In Sect. 3, we linearise the model to study spatial instability of the Maxwellian equilibrium, and derive an explicit instability criterion when the density parameter $\nu$ is small. We then study the perturbative effect of the collision integral, and show that its effect is uniformly small, thus validating the accuracy of the approximate stability criterion. Finally, in the concluding Sect. 4, we offer some conjectures about the subsequent nonlinear evolution of the system.

## The Boltzmann Equation

The basic equation of statistical mechanics is the Liouville equation, from which we can derive the BBGKY hierarchy:

2.1 $$\begin{aligned} \frac{\partial {f_s}}{\partial {t}}+\sum _{i=1}^s\left[ \mathbf{v}_i\cdot {\varvec{ \nabla }}_{\mathbf{r}_i}f_s+\left\{ \mathbf{g}+\sum _{j=1}^s\mathbf{a}_{ij}\right\} \cdot {\varvec{ \nabla }}_{\mathbf{v}_i}f_s\right] =-\sum _{i=1}^s\int _{P}{} \mathbf{a}_{i,s+1}\cdot {\varvec{ \nabla }}_{\mathbf{v}_i}f_{s+1}\,d\gamma _{s+1}.\nonumber \\ \end{aligned}$$

Here $f_s$ is the *s*-particle distribution function, and is a function of the positions $\mathbf{r}_i\in V$ and velocities $\mathbf{v}_i\in U$ of the *s* particles, as well as time *t*,  $P=V\times U$ is the six-dimensional configuration space of position and velocity, and $d\gamma _i$ is the volume element of that space, $\mathbf{g}$ is the external acceleration (typically gravity), and $\mathbf{a}_{ij}$ is the inter-particle acceleration on particle *i* due to particle *j*. If the inter-particle potential is $W_{ij}=W(|\mathbf{r}_i-\mathbf{r}_j|),$ then

2.2 $$\begin{aligned} \mathbf{a}_{ij}=-\dfrac{1}{m}{\varvec{ \nabla }}_{\mathbf{r}_i}W_{ij}. \end{aligned}$$

A typical example of such a potential is the Lennard-Jones potential given by

2.3 $$\begin{aligned} W=W_0\left[ \left( \dfrac{d}{r}\right) ^{12}-\left( \dfrac{d}{r}\right) ^{6}\right] , \end{aligned}$$

where *d* is the molecular diameter; the potential is portrayed in Fig. 1.

**Fig. 2:**
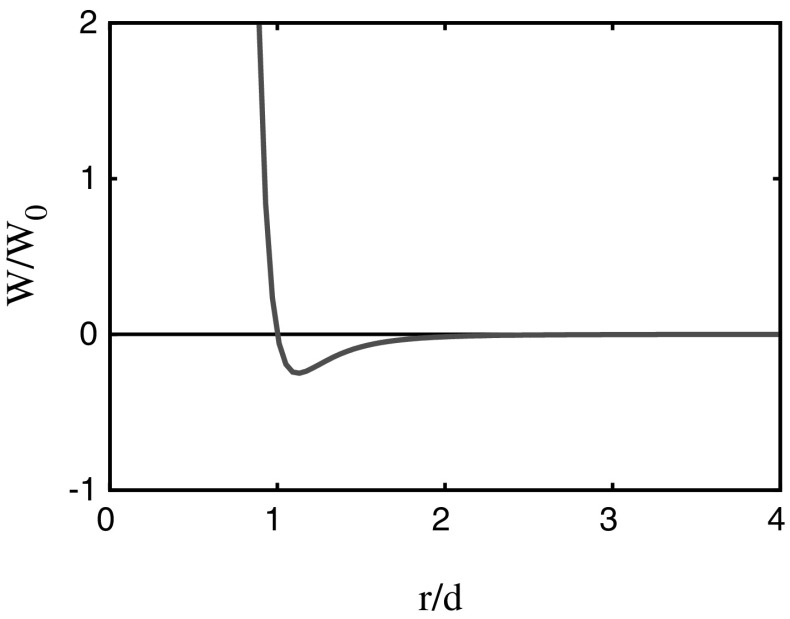
The Lennard-Jones potential for inter-particle forces, as given by (2.3)

Particularly, the one-particle distribution function $f_1=f(\mathbf{r},\mathbf{v},t)$ satisfies the first equation in the hierarchy:

2.4 $$\begin{aligned} \frac{\partial {f}}{\partial {t}}+\mathbf{v}\cdot {\varvec{ \nabla }}_{\mathbf{r}}f+\mathbf{g}\cdot {\varvec{ \nabla }}_{\mathbf{v}}f=-\int _{P}{} \mathbf{a}\cdot {\varvec{ \nabla }}_{\mathbf{v}}f_{2}\,d\gamma _{2}, \end{aligned}$$

where $\mathbf{a}=\mathbf{a}_{12}.$ The Boltzmann equation now follows from the assumption that the locations of two particles in *P* are independent of each other, so that we take

2.5 $$\begin{aligned} f_2(\mathbf{r},\mathbf{v};\mathbf{s},\mathbf{w};t)=f(\mathbf{r},\mathbf{v},t)f(\mathbf{s},\mathbf{w},t), \end{aligned}$$

and in this case we obtain

2.6 $$\begin{aligned} \frac{\partial {f}}{\partial {t}}+\mathbf{v}\cdot {\varvec{ \nabla }}_{\mathbf{r}}f+\mathbf{g}\cdot {\varvec{ \nabla }}_{\mathbf{v}}f=Q, \end{aligned}$$

where the collision integral takes the form

2.7 $$\begin{aligned} Q=-\int _{P}{} \mathbf{a}(\mathbf{r}-\mathbf{s})\cdot {\varvec{ \nabla }}_{\mathbf{v}}f(\mathbf{r},\mathbf{v},t)f(\mathbf{s},\mathbf{w},t)\,d\mathbf{s}\,d\mathbf{w}. \end{aligned}$$

### Intermolecular Forces

Henceforth we ignore the external body force, thus $\mathbf{g}=\mathbf{0}.$ We are now motivated by the form of the potential in Fig. 1 to consider *W* to consist of two parts: an increasing part for $r>d$ corresponding to long range attractive forces, and a vertical line at $r=d$ which corresponds to collisions between particles. In more detail, we consider $W=W_\mathrm{C}(r)+W_\mathrm{LR}(r),$ with both potentials being zero for $r<d,$ and $W_\mathrm{LR}<0$ is an increasing function, tending to 0 as $r\rightarrow \infty ,$ while the collisional potential $W_\mathrm{C}$ is 0 for $r>d,$ but ranges from 0 to $\infty$ at $r=d,$ representing perfectly elastic collision. In this case, the collision integral can be taken to be the sum of two terms,

2.8 $$\begin{aligned} Q=Q_\mathrm{C}+Q_\mathrm{LR}, \end{aligned}$$

where $Q_\mathrm{C}$ is the usual Boltzmann form of the collision integral representing perfectly elastic collisions,

2.9 $$\begin{aligned}&Q_\mathrm{C}=\int _U\int _{\Omega _+}[f(\mathbf{r},\mathbf{v}',t)f(\mathbf{r},\mathbf{w}',t)-f(\mathbf{r},\mathbf{v},t)f(\mathbf{r},\mathbf{w},t)]\,d\Omega \,d\mathbf{w},\nonumber \\&\mathbf{v}'=\mathbf{v}+(\mathbf{V}\cdot \hat{\mathbf{k}})\hat{\mathbf{k}},\quad \mathbf{w}'=\mathbf{w}-(\mathbf{V}\cdot \hat{\mathbf{k}})\hat{\mathbf{k}},\quad \mathbf{V}=\mathbf{w}-\mathbf{v}; \end{aligned}$$

here $U={\mathbf{R}}^3$ is velocity space, $\mathbf{v}$ and $\mathbf{w}$ are the velocities of two colliding particles, $\mathbf{V}$ their relative velocity, $\hat{\mathbf{k}}$ a unit vector along the line between their centres at the point of collision, and $d\Omega =d^2\mathbf{V}\cdot \hat{\mathbf{k}}\,d\omega ,$ with the solid angle element $d\omega$ being taken over all solid angle subtended at $\mathbf{v}$ such that $\mathbf{V}\cdot \hat{\mathbf{k}}>0$ (so that the particles are actually colliding, not separating). A more accurate expression due to Enskog allows for the finite size of the particles, but this is not necessary in the present discussion. Further discussion of the Enskog modification is provided in Sect. 4. $Q_\mathrm{C}$ is associated with the vertical (repulsive) part of the potential, while $Q_\mathrm{LR}$ represents the longer range attractive force,

2.10 $$\begin{aligned} Q_\mathrm{LR}=-\mathbf{A}\cdot {\varvec{ \nabla }}_{\mathbf{v}}f, \end{aligned}$$

where for an inter-particle potential as in (2.2), we have from (2.7)

2.11 $$\begin{aligned} \mathbf{A}=-\int _V\frac{a(\xi ){\varvec{ \xi }}}{\xi }n(\mathbf{r}-{\varvec{ \xi }},t)\,d{\varvec{ \xi }}, \end{aligned}$$

in which

2.12 $$\begin{aligned} n=\int _U f\,d\mathbf{v}\end{aligned}$$

is the number density,

2.13 $$\begin{aligned} a(\xi )=\frac{W'(\xi )}{m} \end{aligned}$$

is the acceleration associated with the attractive part of the potential [thus we take $$a=0$$ for $$\xi <d$$: equivalently, the integral in (2.11) is taken over $${\varvec{ \xi }}\in {\mathbf{R}}^3,$$$$\xi >d$$], and *V* is spatial volume. As mentioned above, the integral in (2.9) over $$\Omega _+$$ is with respect to solid angle in velocity space. Specifically, as shown in Fig. 2, integration is over the solid angle subtended at $$\mathbf{v}$$ over the antipodal sphere through the antipodal points $$\mathbf{v}$$ and $$\mathbf{w},$$ and more precisely

2.14 $$\begin{aligned} d\Omega =d^2\hat{\mathbf{k}}\cdot \mathbf{V}\,d\omega (\hat{\mathbf{k}})=\frac{d^2\,dS}{V}, \end{aligned}$$

where *d* is molecular diameter, $d\omega$ is the element of solid angle indicated in Fig. 2, $\mathbf{V}=\mathbf{w}-\mathbf{v},$*dS* is the element of surface area, and $\hat{\mathbf{k}}$ is the unit vector in the direction of $\mathbf{v}'-\mathbf{v}.$ Note that $d\Omega$ has units of velocity. For the Lennard-Jones potential in (2.3), we would take

2.15 $$\begin{aligned} a(\xi )=\frac{6W_0d^6}{mr^7},\quad r>d, \end{aligned}$$

and this will be assumed later [after (3.14)]; for now $a(\xi )$ is quite general. The Boltzmann equation (2.6) with $\mathbf{g}=\mathbf{0}$ thus takes the form

2.16 $$\begin{aligned} \frac{\partial {f}}{\partial {t}}+\mathbf{v}\cdot {\varvec{ \nabla }}f+\mathbf{A}\cdot {\varvec{ \nabla }}_{\mathbf{v}}f=Q_\mathrm{C}, \end{aligned}$$

and we write ${\varvec{ \nabla }}_{\mathbf{r}}={\varvec{ \nabla }}.$ This equation forms the basis of our study.

**Fig. 3:**
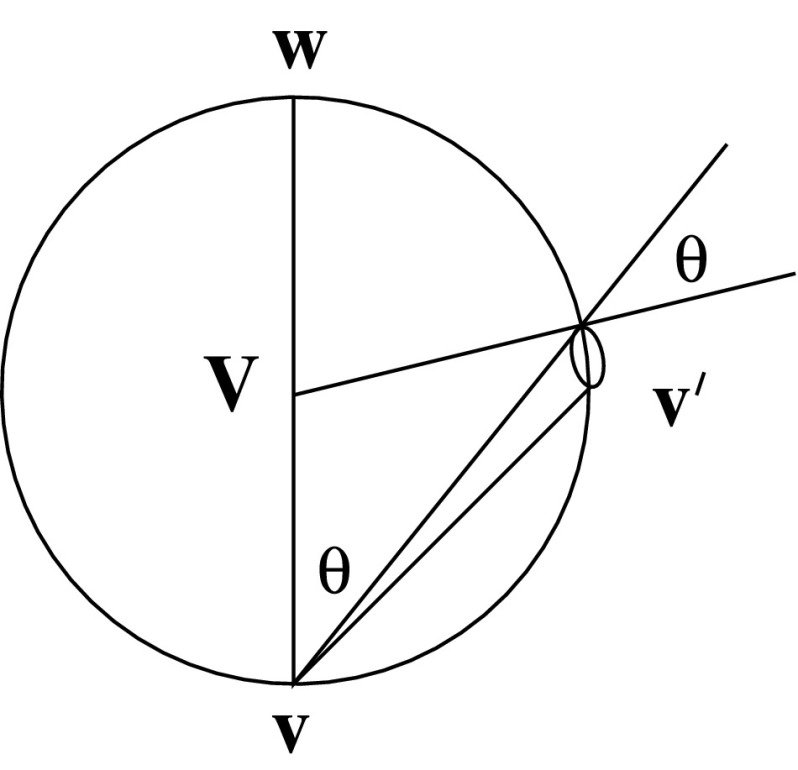
Integration with respect to (scaled) solid angle $\Omega$ over the antipodal sphere

### Non-dimensionalisation

It is convenient to non-dimensionalise the model. To do this, we define the thermal velocity scale

2.17 $$\begin{aligned} v_0=\sqrt{\frac{kT_0}{m}}, \end{aligned}$$

an acceleration scale

2.18 $$\begin{aligned} a_0=\frac{W_0}{md}, \end{aligned}$$

and a mean inter-molecular distance

2.19 $$\begin{aligned} l=\frac{1}{n_0^{1/3}}, \end{aligned}$$

where $n_0$ is a typical value of the number density. We then scale the variables^1^ as

2.20 $$\begin{aligned}&n\sim n_0,\quad f\sim \frac{n_0}{v_0^3},\quad \mathbf{r}\sim l,\quad a=a_0a^*\left( \frac{\xi }{d}\right) ,\nonumber \\&v\sim v_0,\quad t\sim \frac{l}{v_0},\quad \mathbf{A}\sim \frac{a_0d^4}{l^4},\quad Q_\mathrm{C}=\frac{n_0^2d^2}{v_0^2}Q, \end{aligned}$$

and this leads us to the non-dimensional form of the equation,

2.21 $$\begin{aligned}&\frac{\partial {f}}{\partial {t}}+\mathbf{v}\cdot {\varvec{ \nabla }}f+\beta \mathbf{A}\cdot {\varvec{ \nabla }}_{\mathbf{v}}f=\nu ^2Q,\nonumber \\&Q=\int _U\int _{\Omega _+}[f(\mathbf{r},\mathbf{v}',t)f(\mathbf{r},\mathbf{w}',t)-f(\mathbf{r},\mathbf{v},t)f(\mathbf{r},\mathbf{w},t)]\,d\Omega \,d\mathbf{w},\nonumber \\&\mathbf{A}=-\frac{1}{\nu }\int _V\frac{a(\xi ){\varvec{ \xi }}}{\xi }n(\mathbf{r}-\nu {\varvec{ \xi }},t)\,d{\varvec{ \xi }}, \end{aligned}$$

in which all the variables are dimensionless (in particular $d\Omega =dS/V$), we have dropped the asterisk from $a^*,$ and the dimensionless parameters are defined by

2.22 $$\begin{aligned} \beta =\frac{a_0d^4}{v_0^2l^3},\quad \nu =\frac{d}{l}. \end{aligned}$$

This form of scaling the equation is that used by Keller [14], although in his case the interpretation of the parameter $\nu$ is rather different.

In the absence of any spatial variation, $\mathbf{A}=\mathbf{0}$ and the left hand side of the equation is simply $\partial f/\partial t.$ As is well known, the existence of Boltzmann’s *H*-function assures us that *f* approaches an equilibrium on a time scale of $O\left( \dfrac{1}{\nu ^2}\right) ,$ and this is the Maxwellian distribution, which in its dimensionless form is given by

2.23 $$\begin{aligned} f=f_0(v)=\frac{1}{(2\pi )^{3/2}}e^{-\frac{1}{2}v^2}, \end{aligned}$$

where for convenience we will assume that the mean velocity is zero. It is the stability of this state to spatial perturbations which is our concern.

## Linear Stability

Before we linearise the equation, it is convenient first to define

3.1 $$\begin{aligned} f=e^\Phi , \end{aligned}$$

so that (2.21)$_1$ takes the form

3.2 $$\begin{aligned} \frac{\partial {\Phi }}{\partial {t}}+\mathbf{v}\cdot {\varvec{ \nabla }}\Phi +\beta \mathbf{A}\cdot {\varvec{ \nabla }}_{\mathbf{v}}\Phi =\nu ^2\int _U\int _{\Omega _+}[\exp (\Delta \Phi )-1])f(\mathbf{r},\mathbf{w},t)\,d\Omega \,d\mathbf{w}, \end{aligned}$$

where for $$\psi (\mathbf{v}),$$

3.3 $$\begin{aligned} \Delta \psi =\psi (\mathbf{v}')+\psi (\mathbf{w}')-\psi (\mathbf{v})-\psi (\mathbf{w}). \end{aligned}$$

The Maxwellian corresponds to

3.4 $$\begin{aligned} \Phi _0=-\tfrac{1}{2}v^2-\tfrac{3}{2}\ln (2\pi ), \end{aligned}$$

and $$\Delta \Phi _0=0.$$

We now linearise the equation about the steady state by writing

3.5 $$\begin{aligned} \Phi =\Phi _0+\phi , \end{aligned}$$

and neglecting nonlinear terms; the linearised form of (3.2) is

3.6 $$\begin{aligned} \phi _t+\mathbf{v}\cdot {\varvec{ \nabla }}\phi -\beta \mathbf{A}\cdot \mathbf{v}=\nu ^2\mathcal{L}\phi , \end{aligned}$$

where the linearised form of $\mathbf{A}$ is

3.7 $$\begin{aligned} \mathbf{A}=-\frac{1}{\nu }\int _P\frac{a(\xi ){\varvec{ \xi }}}{\xi }f_0(v)\phi (\mathbf{r}-\nu {\varvec{ \xi }},\mathbf{v},t)\,d\mathbf{v}\,d{\varvec{ \xi }}, \end{aligned}$$

and the linearised collision operator is

3.8 $$\begin{aligned} \mathcal{L}\phi =\int _U\int _{\Omega _+}f_0(w)\Delta \phi \,d\Omega \,d\mathbf{w}. \end{aligned}$$

We now seek normal mode solutions to this equation of the form

3.9 $$\begin{aligned} \phi =\psi (\mathbf{v})e^{i\mathbf{k}\cdot \mathbf{r}+\sigma t}, \end{aligned}$$

where $\mathbf{k}$ is the wave vector. This leads to the eigenvalue problem for $\psi (\mathbf{v})$ in the form

3.10 $$\begin{aligned} (\sigma +i\mathbf{k}\cdot \mathbf{v})\psi -\beta \mathbf{B}\cdot \mathbf{v}=\nu ^2\mathcal{L}\psi , \end{aligned}$$

where

3.11 $$\begin{aligned} \mathbf{B}=-\frac{1}{\nu }\int _V\frac{a(\xi ){\varvec{ \xi }}}{\xi }e^{-i\nu \mathbf{k}\cdot {\small {\varvec{ \xi }}}}\,d{\varvec{ \xi }}, \end{aligned}$$

and we have assumed a normalisation in which

3.12 $$\begin{aligned} \int _Uf_0(v)\psi (\mathbf{v})\,d\mathbf{v}=1. \end{aligned}$$

To evaluate $\mathbf{B},$ we take Cartesian coordinates in ${ V}={\mathbf{R}}^3$ with the *z* axis in the $\mathbf{k}$ direction. By symmetry, the *x* and *y* components are zero, and therefore we can write

3.13 $$\begin{aligned} \mathbf{B}=iC(\nu k)\mathbf{k}, \end{aligned}$$

and by changing to spherical polar coordinates, we find that (writing $K=\nu k$)

3.14 $$\begin{aligned} C(K)=\frac{4\pi }{K^2}\int _1^\infty ra(r)\left[ \frac{\sin Kr}{Kr}-\cos Kr\right] \,dr. \end{aligned}$$

This expression was derived by Liboff [15]. The function *C*(*K*) is plotted in Fig. 3; its asymptotic limits for small and large *K* are [taking $a=6/r^7,$ corresponding to (2.15), and with $a_0$ given in (2.18)]

3.15 $$\begin{aligned}&C\sim \tfrac{8}{3}\pi -\tfrac{4 }{5}\pi K^2+\cdots ,\quad K\rightarrow 0,\nonumber \\&C\sim \frac{24\pi \sin K}{K^3}+\cdots , \quad K\rightarrow \infty . \end{aligned}$$

Some further detail of the expansion for small *K* is given in the Appendix. As a consequence, (3.10) is

3.16 $$\begin{aligned} (\sigma +i\mathbf{k}\cdot \mathbf{v})\psi -i\beta C \mathbf{k}\cdot \mathbf{v}=\nu ^2\mathcal{L}\psi . \end{aligned}$$

Our aim is to solve this equation to determine $\sigma (\mathbf{k}).$ Eigenfunctions $\psi$ for which $\mathrm{Re\,}\sigma >0$ are unstable.

**Fig. 4:**
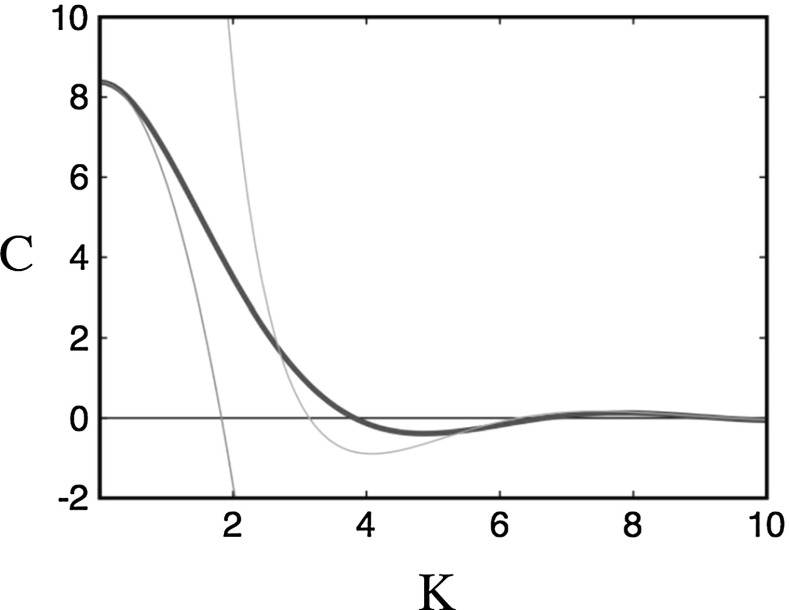
The function $C(\nu k)=C(K)$ defined by (3.14). The thin lines give the asymptotic limits from (3.15)

### The Limit $\nu \rightarrow 0$

Before we begin, we summarise some of what is known about the linear integral operator $\mathcal{L}$ (e. g., Cercignani [7], p. 159 ff). Under the definition of an inner product

3.17 $$\begin{aligned} \langle \phi ,\psi \rangle =\int _Uf_0(\mathbf{v})\phi (\mathbf{v})\bar{\psi }(\mathbf{v})\,d\mathbf{v}, \end{aligned}$$

$\mathcal{L}$ is self-adjoint. Its null-space $\mathcal{N}$ is the span of the functions 1,  $\mathbf{v}$ and $\tfrac{1}{2}v^2;$ all other eigenfunctions have negative eigenvalues. Something is known about the action of $\mathcal{L}$ on tensors; for example,

3.18 $$\begin{aligned} \mathcal{L}[g(v)\mathbf{v}]=h(v)\mathbf{v},\quad \mathcal{L}\left[ G(v)\left\{ \mathbf{v}\mathbf{v}-\tfrac{1}{3}{\varvec{ \delta }}\right\} \right] =H(v)\left[ \mathbf{v}\mathbf{v}-\tfrac{1}{3}{\varvec{ \delta }}\right] , \end{aligned}$$

and such results can be extended to higher order tensors; they are due to a rotational invariance of the operator $\mathcal{L}.$ However, the ability to directly solve an equation such as (3.16), despite all this structure, is not clear, and we therefore resort to an approximate method.

Since $\beta \propto v_0^{-1}\propto T_0^{-1/2},$ we would hope that as the ambient temperature decreases, the instability that we seek will appear at a sufficiently large value of $\beta .$ On the other hand, while molecular spacings in liquids are of order *d*,  those in gases are significantly larger (because gas densities are typically much lower at normal temperatures and pressures).^2^ This suggests that we consider a gas in which $l\gg d,$ and thus $\nu \ll 1.$ This immediately allows us to provide an approximate solution of (3.16). The neglect of the collision integral requires consideration, in case it allows a singular perturbation; in the sequel we give some consideration to this possibility, although we might suspect, since the neglected term is an integral, that the induced perturbation is in fact regular.

If we neglect the term of $O(\nu ^2)$ in (3.16), we simply have

3.19 $$\begin{aligned} \psi =\frac{i\beta C \mathbf{k}\cdot \mathbf{v}}{\sigma +i\mathbf{k}\cdot \mathbf{v}}, \end{aligned}$$

and the normalisation condition (3.12) implies

3.20 $$\begin{aligned} i\beta C\int _U\frac{f_0(v)\mathbf{k}\cdot \mathbf{v}\,d\mathbf{v}}{\sigma +i\mathbf{k}\cdot \mathbf{v}}=1, \end{aligned}$$

and it is this which determines $\sigma .$

To evaluate (3.20), we take Cartesian axes $(v_x,v_y,\zeta )$ in the velocity space *U*,  with the $\zeta$ axis in the direction of $\mathbf{k},$ so that $\mathbf{k}\cdot \mathbf{v}=k{\zeta }.$ Carrying out the integrals in ${ v_x}$ and ${ v_y},$ this leads to

3.21 $$\begin{aligned} \frac{i\beta C}{\sqrt{2\pi }}\int _{-\infty }^\infty \frac{k{\zeta }\,e^{-\frac{1}{2}{\zeta }^2}\,d{\zeta }}{\sigma +ik{\zeta }}=1. \end{aligned}$$

Defining

3.22 $$\begin{aligned} \sigma =\sqrt{2}k\eta , \end{aligned}$$

this can be manipulated to the form

3.23 $$\begin{aligned} \frac{1}{\beta C}=\frac{1}{\sqrt{\pi }}\int _{-\infty }^\infty \frac{{ z}e^{-{ z}^2}\,d{ z}}{{ z}-i\eta }, \end{aligned}$$

and then

3.24 $$\begin{aligned} 1-\frac{1}{\beta C}=\frac{\eta }{i\sqrt{\pi }}\int _{-\infty }^\infty \frac{e^{-{ z}^2}\,d{ z}}{{ z}-i\eta }=\frac{2\eta ^2}{\sqrt{\pi }}\int _{0}^\infty \frac{e^{-{ z}^2}\,d{ z}}{{ z}^2+\eta ^2}. \end{aligned}$$

This integral is related to a form of the ‘plasma dispersion function’ (Fettis et al. [12], Abramowitz and Stegun [1], p. 297). Specifically, if we define

3.25 $$\begin{aligned} w(i\eta )\equiv W(\eta )=e^{\eta ^2}\mathrm{erfc\,}\eta , \end{aligned}$$

then, providing $\mathrm{Re\,}\eta >0,$ the integral in (3.24) is proportional to $W(\eta ),$ and

3.26 $$\begin{aligned} 1-\frac{1}{\beta C}=\sqrt{\pi }\eta W(\eta )=\sqrt{\pi }\eta e^{\eta ^2}\mathrm{erfc\,}\eta . \end{aligned}$$

If $\eta$ is a root of (3.23), then so is $-\bar{\eta }$ (by taking the complex conjugate of the equation): it follows that we can take $\mathrm{Re\,}\eta \ge 0$ without loss of generality, since for $\mathrm{Re\,}\eta <0,$ we simply consider $-\bar{\eta }.$ By taking the imaginary part of the second integral in (3.24), it follows that in fact either $\eta$ is real or purely imaginary. The latter case corresponds to neutral stability, and is discussed later. For real $\eta ,$ we can then suppose $\eta >0.$ In this case, (3.24) shows that a root only exists if $\beta C>1,$ and this is shown in Fig. 4.

This is the principal result that we obtain: for values $\beta C>1,$ the uniform state is unstable, and we associate this with a transition to condensation. With $C(\nu k)$ as shown in Fig. 3, we see that the criterion for instability is that

3.27 $$\begin{aligned} \beta >\frac{3}{8\pi }, \end{aligned}$$

or in dimensional terms,

3.28 $$\begin{aligned} T_0<T_c,\quad T_c=\frac{d^2}{km^{1/3}}\left( \frac{8\pi W_0p}{3}\right) ^{2/3}, \end{aligned}$$

where for illustrative purposes we have used the perfect gas law $p=n_0kT_0,$ even though this may not be appropriate for a Boltzmann–Vlasov gas.

We can see from Fig. 3 that in the typical case where $\beta \sim O(1)$ (and $\beta >\frac{3}{8\pi }$), $\beta C$ decreases to one as *k* increases from zero to a value of $O(1/\nu ),$ and thus Fig. 4 shows that $\eta$ decreases monotonically to zero as *k* increases. Hence $\sigma$ is a concave unimodal function which first increases from zero and then decreases back to zero. In particular, the maximum growth rate occurs for $k\sim \dfrac{1}{\nu },$ and is of order $\dfrac{1}{\nu }.$

Instability first sets in when $\beta$ increases through $\frac{3}{8\pi },$ and near this value, long wavelength disturbances are amplified since $K\ll 1.$ We use the expansion in (3.15) for small *K*,  and expand (3.26) for small $\eta ,$ and this leads to the approximation

3.29 $$\begin{aligned} \sigma \approx \sqrt{\frac{2}{\pi }}k\left[ \left( \tfrac{8}{3}\beta \pi -1\right) -\tfrac{3}{10}\nu ^2k^2\right] , \end{aligned}$$

for which the maximum growth rate occurs at

3.30 $$\begin{aligned} k\approx \frac{1}{3\nu }\left[ 10\left( \tfrac{8}{3}\beta \pi -1\right) \right] ^{1/2}. \end{aligned}$$

In practice, it is only such long wavelength modes which are of interest.

**Fig. 5:**
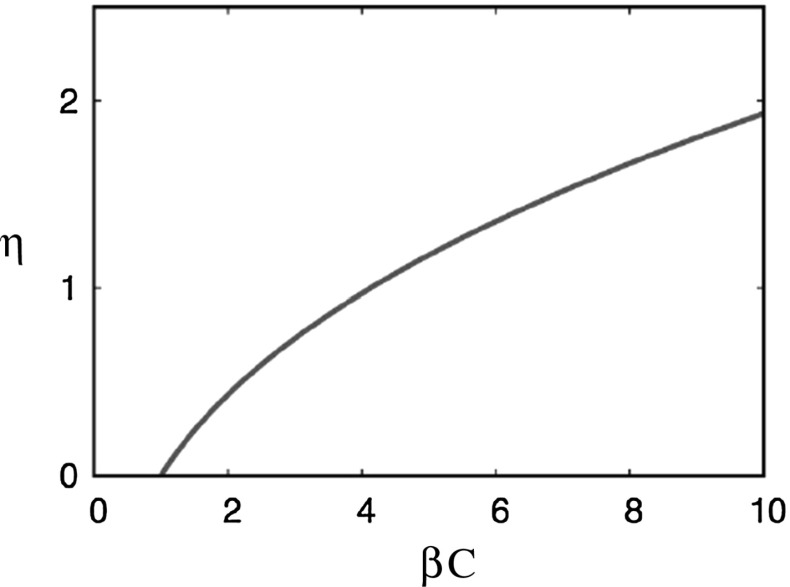
The scaled growth rate $\eta =\dfrac{\sigma }{\sqrt{2}k}$ as a function of $\beta C,$ given by (3.26)

### An Approximate Correction

We now turn to the consideration of the accuracy of this stability criterion. The issue is whether the neglect of the collision integral term $\nu ^2\mathcal{L}\psi$ in (3.16) is justified. Taking (3.19) as our definition of $\psi ,$ we have

3.31 $$\begin{aligned} \psi (\mathbf{v}')-\psi (\mathbf{v})=\frac{i\beta C\sigma \mathbf{k}\cdot (\mathbf{v}'-\mathbf{v})}{(\sigma +i\mathbf{k}\cdot \mathbf{v}')(\sigma +i\mathbf{k}\cdot \mathbf{v})}, \end{aligned}$$

and it follows that

3.32 $$\begin{aligned} |\psi (\mathbf{v}')-\psi (\mathbf{v})|\le \frac{\beta C\sigma k|\mathbf{v}'-\mathbf{v}|}{\{\sigma ^2+(\mathbf{k}\cdot \mathbf{v}')^2\}^{1/2}\{\sigma ^2+(\mathbf{k}\cdot \mathbf{v})^2\}^{1/2}}\le \frac{\beta Ck|\mathbf{v}'-\mathbf{v}|}{\{\sigma ^2+(\mathbf{k}\cdot \mathbf{v})^2\}^{1/2}}.\nonumber \\ \end{aligned}$$

It can be seen that the concession admitted in the last inequality is quite weakening, but we have not found any easy way round this. In a similar way we have

3.33 $$\begin{aligned} |\psi (\mathbf{w}')-\psi (\mathbf{w})|\le \frac{\beta Ck|\mathbf{v}'-\mathbf{v}|}{\{\sigma ^2+(\mathbf{k}\cdot \mathbf{w})^2\}^{1/2}}, \end{aligned}$$

using the fact that $\mathbf{w}'-\mathbf{w}=-(\mathbf{v}'-\mathbf{v}).$ It follows that

3.34 $$\begin{aligned} |\Delta \psi |\le \beta Ck\left[ \frac{1}{\{\sigma ^2+(\mathbf{k}\cdot \mathbf{v})^2\}^{1/2}} +\frac{1}{\{\sigma ^2+(\mathbf{k}\cdot \mathbf{w})^2\}^{1/2}}\right] |\mathbf{v}'-\mathbf{v}|, \end{aligned}$$

and thus that

3.35 $$\begin{aligned} |\mathcal{L}\psi |\le \beta Ck\int _Uf_0(w)\left[ \frac{1}{\{\sigma ^2+(\mathbf{k}\cdot \mathbf{v})^2\}^{1/2}}+\frac{1}{\{\sigma ^2+(\mathbf{k}\cdot \mathbf{w})^2\}^{1/2}}\right] \int _{\Omega _+}|\mathbf{v}'-\mathbf{v}|\,d\Omega \,d\mathbf{w}.\nonumber \\ \end{aligned}$$

The integral with respect to $\Omega$ can be evaluated (using the geometry of Fig. 2), and is $\tfrac{2}{3}\pi V^2,$ where $V=|\mathbf{v}-\mathbf{w}|.$ Therefore

3.36 $$\begin{aligned} |\mathcal{L}\psi |\le & {} \tfrac{2}{3}\pi \beta Ck\int _Uf_0(w)|\mathbf{v}-\mathbf{w}|^2\left[ \frac{1}{\{\sigma ^2+(\mathbf{k}\cdot \mathbf{v})^2\}^{1/2}}+\frac{1}{\{\sigma ^2+(\mathbf{k}\cdot \mathbf{w})^2\}^{1/2}}\right] \,d\mathbf{w}\nonumber \\= & {} \tfrac{2}{3}\pi \beta Ck\int _Uf_0(w)(v^2+w^2)\left[ \frac{1}{\{\sigma ^2+(\mathbf{k}\cdot \mathbf{v})^2\}^{1/2}}+\frac{1}{\{\sigma ^2+(\mathbf{k}\cdot \mathbf{w})^2\}^{1/2}}\right] \,d\mathbf{w},\nonumber \\ \end{aligned}$$

where the integral proportional to $\mathbf{v}\cdot \mathbf{w}$ vanishes due to symmetry considerations. The integrals can be evaluated, and this leads finally to

3.37 $$\begin{aligned} |\mathcal{L}\psi |\le \tfrac{1}{3}\beta Ck\left[ \frac{v^2+3}{\{\sigma ^2+(\mathbf{k}\cdot \mathbf{v})^2\}^{1/2}}+\frac{1}{k\sqrt{2\pi }}\left\{ (v^2+2)e^\xi K_0(\xi )-2\xi [e^\xi K_0(\xi )]'\right\} \right] ,\nonumber \\ \end{aligned}$$

where

3.38 $$\begin{aligned} \xi =\frac{\sigma ^2}{4k^2}. \end{aligned}$$

Thus our upper bound implies $|\mathcal{L}(\psi )|\le O(v^2),$ which would imply that the neglect of the collision term in (3.16) becomes invalid when $v\sim \dfrac{1}{\nu ^2}.$ We surmise, however, that in fact this is not the case, and that actually $|\mathcal{L}(\psi )|\le O(v).$

Our reasons for this surmise follow from consideration of $$\mathcal{L}\psi $$ when *v* is large. Because the Maxwellian $$f_0$$ decays rapidly as *w* increases, the integral over *U* in the definition of $$\mathcal{L}$$ is only significant when $$w\sim 1.$$ The integral over $$\Omega _+$$ thus splits into two parts: a region near the origin where $$dS\sim 1$$ and thus $$d\Omega \sim 1/V,$$ where either $$v'$$ or $$w'\sim 1,$$$$\Delta \psi \sim 1,$$ and this part of the integral is *O*(1 / *V*). Over the rest of $$\Omega _+,$$$$v'\sim w'\sim v,$$

3.39 $$\begin{aligned} \Delta \psi =\frac{\beta C\sigma }{\sigma +i\mathbf{k}\cdot \mathbf{w}}+O\left( \frac{1}{V}\right) , \end{aligned}$$

and thus we have (since $\int _{\Omega _+}d\Omega =\pi V$)

3.40 $$\begin{aligned} \mathcal{L}\psi =\pi \beta C\sigma \int _U\frac{|\mathbf{v}-\mathbf{w}|f_0(w)\,d\mathbf{w}}{\sigma +i\mathbf{k}\cdot \mathbf{w}}+O(1), \end{aligned}$$

and is thus of *O*(*v*): specifically,

3.41 $$\begin{aligned} \mathcal{L}\psi \approx \pi \beta C\sigma v\int _U\frac{f_0(w)\,d\mathbf{w}}{\sigma +i\mathbf{k}\cdot \mathbf{w}}=\frac{\pi \beta C\sigma v}{\sqrt{2\pi }}\int _{-\infty }^\infty \frac{e^{-\frac{1}{2}{\zeta }^2}\,d{\zeta }}{\sigma +ik{\zeta }}=\pi ^{3/2}\beta Cv\eta e^{\eta ^2}\mathrm{erfc\,}\eta ,\nonumber \\ \end{aligned}$$

where we take the ${\zeta }$ axis in the direction of $\mathbf{k},$ and $\eta$ is still defined through (3.22). In view of (3.26), this is simply

3.42 $$\begin{aligned} \mathcal{L}\psi \approx \pi (\beta C-1)v, \end{aligned}$$

and allows a correction to (3.19) as

3.43 $$\begin{aligned} \psi =\frac{i\beta C \mathbf{k}\cdot \mathbf{v}+\nu ^2\pi (\beta C-1)v}{\sigma +i\mathbf{k}\cdot \mathbf{v}}, \end{aligned}$$

and this could be used to provide an improved estimate for $\sigma ;$ the main point, however, is that the collision term appears to provide a regular correction to our leading order result.

### Wave Motion

The question arises, of course, as to what happens if $\beta C<1.$ In this case, the only possibility is a pure wave motion in which $\sigma =-i\omega ,$ and in that case the defining equation for $\psi ,$ (3.16), can be written as

3.44 $$\begin{aligned} (\mathbf{k}\cdot \mathbf{v}-\omega )\psi -\beta C\mathbf{k}\cdot \mathbf{v}={ 0}, \end{aligned}$$

where again we neglect the collision term on the basis that $\nu$ is small , and we again normalise $\psi$ by (3.12), that is,

3.45 $$\begin{aligned} \int _Uf_0(v)\psi (\mathbf{v})\,d\mathbf{v}=1. \end{aligned}$$

The solution of (3.44) is singular,

3.46 $$\begin{aligned} \psi =\frac{\beta C\mathbf{k}\cdot \mathbf{v}}{\mathbf{k}\cdot \mathbf{v}-\omega }, \end{aligned}$$

and it is necessary to enquire how one should interpret the resulting integral in (3.45). One way to do this is to consider (3.46) as the limit as $\varepsilon \rightarrow 0$ of the non-singular solution

3.47 $$\begin{aligned} \psi =\frac{\beta C\mathbf{k}\cdot \mathbf{v}}{\mathbf{k}\cdot \mathbf{v}-\omega -i\varepsilon k}, \end{aligned}$$

corresponding to $\sigma =\varepsilon k-i\omega .$ With $\mathbf{v}=(v_x,v_y,\zeta ),$ and carrying out the integral with respect to $v_x$ and $v_y$ (and with $\zeta$ in the direction of $\mathbf{k}$), (3.45) leads to

3.48 $$\begin{aligned} \frac{\beta C}{\sqrt{2\pi }}\int _{-\infty }^\infty \frac{\zeta e^{-\frac{1}{2}\zeta ^2}\,d\zeta }{\zeta -c-i\varepsilon }=1, \end{aligned}$$

where $c=\omega /k$ is the wave speed. If we take $\varepsilon >0,$ then we can replace the contour in (3.48) by one which is indented by a lower semi-circle of radius $\delta \ll 1.$ We then let $\varepsilon \rightarrow 0,$ and subsequently $\delta \rightarrow 0,$ and this leads to taking the limit of (3.48) as3.49which is equivalent to interpreting (3.46) as

3.50 $$\begin{aligned} \psi =\beta C\left[ \mathcal{P}\left( \frac{\mathbf{k}\cdot \mathbf{v}}{\mathbf{k}\cdot \mathbf{v}-\omega }\right) +i\pi \mathbf{k}\cdot \mathbf{v}\,\delta (\mathbf{k}\cdot \mathbf{v}-\omega )\right] , \end{aligned}$$

where $\mathcal{P}$ indicates that principal value integration should be applied in (3.45), and we have used the result $|a|\delta (at)=\delta (t).$

However, (3.49) evidently has no solution. What has gone wrong? The problem is that the singularity of (3.44) allows the more general solution to be

3.51 $$\begin{aligned} \psi =\frac{\beta C\mathbf{k}\cdot \mathbf{v}}{\mathbf{k}\cdot \mathbf{v}-\omega }+\sqrt{2\pi }A\,\delta \!\left( {\zeta }-\frac{\omega }{k}\right) , \end{aligned}$$

where *A* is an arbitrary constant, since in the sense of generalised functions, $t\delta (t)=0$ (Carrier et al. [5], p. 320). This situation occurs in a number of other examples. Particularly, we may think of the (two-dimensional) stability of inviscid Couette flow [6], which can be described by the eigenvalue problem

3.52 $$\begin{aligned} (ky-\omega )(\phi ''-k^2\phi )=0, \end{aligned}$$

with boundary conditions $\phi (0)=\phi (1)=0.$ In the linearised problem, the basic flow is $(u,v)=(y,0),$ and the perturbed stream function is given by the normal mode solution

3.53 $$\begin{aligned} \psi =\phi (y)\exp [i(kx-\omega t)]. \end{aligned}$$

There are no regular solutions of (3.52) at all (the discrete spectrum is empty), and solutions are obtained from

3.54 $$\begin{aligned} \phi ''-k^2\phi =\delta (y-c), \end{aligned}$$

where $c=\omega /k$ is the (real) wave speed. With $0<c<1,$ the solutions are just the Green’s functions for the differential operator, and they form the continuous spectrum of the problem.^3^ For the instability of more general shear flows (*U*(*y*), 0), the inviscid stability equation is the Rayleigh equation [11]

3.55 $$\begin{aligned} (U-c)(\phi ''-k^2\phi )-U''\phi =0, \end{aligned}$$

and the solutions are still singular

The normalisation condition now leads, after some algebra, to3.56where we have defined

3.57 $$\begin{aligned} \omega =\sqrt{2}k\Omega , \end{aligned}$$

and using the Plemelj formulae together with the definition of *w*(*z*) in (3.24) and (3.25), this leads to the definition of *A* as

3.58 $$\begin{aligned} A=2\beta C\Omega \int ^\Omega _0e^{t^2}\,dt+(1-\beta C)e^{\Omega ^2}. \end{aligned}$$

It may be noted that if $\beta C<1,$ the possibility of $A=0$ is not available. In view of (3.57), the wave speed is $\sqrt{2}\Omega .$

## Discussion and Conclusions

It has been previously shown [15] that the Maxwellian equilibrium distribution of a (rarified) gas is spatially unstable at sufficiently low temperatures, providing the collisional operator can be ignored. Efforts to include collisional effects have been limited to the algebraic BGK approximation [2, 15], or other such simplifications [13]. In this paper, we have followed a different approach analogous to that of Keller [14], where direct asymptotic solutions of the Boltzmann–Vlasov equation are sought. At leading order this reduces the stability problem to that of the collisionless Vlasov problem, but we have additionally shown that the correction due to the collisional term remains small, so that the resulting stability criterion remains accurate.

It remains to consider the nonlinear evolution of the distribution function. This is beyond the scope of the present paper, but the results of de Sobrino [10] and the scaled equation in (2.21) suggest a way forward. First, the closure for the two-particle distribution function in (2.5) is replaced by

4.1 $$\begin{aligned} f_2(\mathbf{r},\mathbf{v};\mathbf{s},\mathbf{w};t)=C\left\{ \tfrac{1}{2}(\mathbf{r}+\mathbf{s}),t\right\} f(\mathbf{r},\mathbf{v},t)f(\mathbf{s},\mathbf{w},t), \end{aligned}$$

where *C* is a crowding coefficient, designed to represent the reduction of available space in dense conditions; de Sobrino suggests

4.2 $$\begin{aligned} C=\frac{1}{1-bn},\quad b=\tfrac{2}{3}\pi d^3. \end{aligned}$$

Second, the collision integral in (2.9) is replaced by the more accurate

4.3 $$\begin{aligned} Q_C=\int _U\int _{\Omega _+}[f_2(\mathbf{r},\mathbf{v}';\mathbf{r}+d\hat{\mathbf{k}},\mathbf{w}';t)-f_2(\mathbf{r},\mathbf{v};\mathbf{r}-d\hat{\mathbf{k}},\mathbf{w};t)]\,d\Omega \,d\mathbf{w}; \end{aligned}$$

hence

4.4 $$\begin{aligned} Q_C= & {} \int _U\int _{\Omega _+}\left[ C\left( \mathbf{r}+\tfrac{1}{2}d\hat{\mathbf{k}},t\right) f(\mathbf{r},\mathbf{v}',t)f(\mathbf{r}+d\hat{\mathbf{k}},\mathbf{w}',t)\dfrac{}{}\right. \nonumber \\&\left. -\ C\left( \mathbf{r}-\tfrac{1}{2}d\hat{\mathbf{k}},t\right) f(\mathbf{r},\mathbf{v},t)f(\mathbf{r}-d\hat{\mathbf{k}},\mathbf{w},t)\right] \,d\Omega \,d\mathbf{w}. \end{aligned}$$

The dimensionless form of this, using (2.20), is

4.5 $$\begin{aligned} Q= & {} \int _U\int _{\Omega _+}\left[ C\left( \mathbf{r}+\tfrac{1}{2}\nu \hat{\mathbf{k}},t\right) f(\mathbf{r},\mathbf{v}',t)f(\mathbf{r}+\nu \hat{\mathbf{k}},\mathbf{w}',t)\dfrac{}{}\right. \nonumber \\&\left. -\ C\left( \mathbf{r}-\tfrac{1}{2}\nu \hat{\mathbf{k}},t\right) f(\mathbf{r},\mathbf{v},t)f(\mathbf{r}-\nu \hat{\mathbf{k}},\mathbf{w},t)\right] \,d\Omega \,d\mathbf{w}, \end{aligned}$$

where from (4.2),

4.6 $$\begin{aligned} C=\frac{1}{1-\tfrac{2}{3}\pi \nu ^3 n}, \end{aligned}$$

and additionally

4.7 $$\begin{aligned}&\frac{\partial {f}}{\partial {t}}+\mathbf{v}\cdot {\varvec{ \nabla }}f+\beta \mathbf{A}\cdot {\varvec{ \nabla }}_{\mathbf{v}}f=\nu ^2Q,\dfrac{}{}\nonumber \\&\mathbf{A}=-\frac{1}{\nu }\int _V\frac{a(\xi ){\varvec{ \xi }}}{\xi }n(\mathbf{r}-\nu {\varvec{ \xi }},t)\,d{\varvec{ \xi }},\dfrac{}{}\nonumber \\&n=\int _Vf\,d\mathbf{v}. \end{aligned}$$

For small $$\nu ,$$ the system reduces to that studied earlier, so that the instability result is the same. It is of at least mathematical interest to ask what state the system then evolves to. Naturally, we would hope that this state would correspond to a condensed liquid, although we recognise that it is generally considered that the Boltzmann (or Boltzmann–Vlasov, or Enskog–Vlasov) equation becomes inapplicable in this case, when the particle spacing is of order *d*. Despite this, it is of interest to enquire whether the solution of (4.7) evolves to a ‘liquid-like’ state. The obvious suggestion is that *f* evolves without the collision term towards a singularity, in which a rescaling becomes necessary when $$f\sim 1/\nu ^3,$$$$r\sim \nu ,$$ and thus $$n\sim 1/\nu ^3,$$$$A\sim 1/\nu ,$$$$t\sim \nu $$ and $$Q\sim 1/\nu ^6,$$ following which the system is described by (4.5) and (4.7), with $$\nu $$ replaced by one everywhere. Exploration of this possibility is postponed to a future study.

## Appendix

The function *C*(*K*) defined in (3.14) can be written also in the form [taking $a(r)=6/r^7$]

A.1 $$\begin{aligned} C(K)=\left[ \frac{4}{K^2}+1-\frac{K^2}{6}\right] \frac{\sin K}{K}+\left[ -\frac{4}{K^2}-\frac{1}{3}+\frac{K^2}{6}\right] \cos K+\frac{\pi K^3}{12}-\mathrm{Si}(K),\nonumber \\ \end{aligned}$$

where

A.2 $$\begin{aligned} \mathrm{Si}(K)=\int _0^K \frac{\sin s\,ds}{s}. \end{aligned}$$

Taylor expansion of this leads to the expression

A.3 $$\begin{aligned} C(K)=\pi \left( \frac{8}{3}-\frac{4K^2}{5}+\frac{\pi K^3}{12}\right) +\sum _2^\infty (-1)^r\pi a_rK^{2r}, \end{aligned}$$

where

A.4 $$\begin{aligned} a_r= & {} -\frac{4}{(2r+3)!}+\frac{4}{(2r+2)!}+\frac{1}{(2r+1)!}+\frac{1}{3(2r)!}\nonumber \\&+\frac{1}{6(2r-1)!}+\frac{1}{6(2r-2)!}-\frac{1}{6(2r-3)(2r-3)!}. \end{aligned}$$

The values of the coefficients $a_2$ to $a_8$ are tabulated in Table  1.

**Table 1:** The values of $a_2$ to $a_8$ given by (A.4)

$a_2$	$-2.85714285714286\times 10^{-2}$
$a_3$	$-1.76366843033503\times 10^{-4}$
$a_4$	$-1.20250120250144\times 10^{-6}$
$a_5$	$-6.6071494642872\times 10^{-9}$
$a_6$	$-2.85494112655234\times 10^{-11}$
$a_7$	$-9.81454168794222\times 10^{-14}$
$a_8$	$-2.73178032811039\times 10^{-16}$

If terms are included out to $O(K^{10}),$ the Taylor series more or less coincides with *C*(*K*) until the point where the large *K* result takes over. The one term expansion at large *K* in (3.15) can be extended by continued integration of parts as

A.5 $$\begin{aligned} C\sim 24\pi \left[ \frac{\sin K}{K^3}\left( 1-\frac{35}{K^2}\ldots \right) -\frac{5\cos K}{K^4}\left( 1-\frac{56}{K^2}\ldots \right) \right] , \end{aligned}$$

but the higher order terms evidently do not become effective until $K\gg 6,$ whereas the first order expression in (3.15) is already accurate for $K\gtrsim 6.$

## Footnotes

1 We have chosen for simplicity to use the particle diameter *d* as the length scale over which the long range forces vary. A more general approach allows *a* to vary over a length scale $d_\mathrm{LR}\gg d,$ and this would allow extra flexibility in the analysis, at the expense of introducing a further dimensionless parameter. But in fact the present analysis includes this more general assumption as a special case.

2 This is not true near the critical point, but we are mainly interested in normal conditions.

3 Almost the same result occurs in the Eady model of baroclinic instability (Pedlosky [17], p. 523).
